# Antibiotics for culture-negative neonatal early-onset sepsis: measuring the unmeasurable

**DOI:** 10.1038/s41390-024-03761-9

**Published:** 2024-12-02

**Authors:** Dustin D. Flannery, Matthew B. Green

**Affiliations:** 1 Division of Neonatology, Children’s Hospital of Philadelphia, Philadelphia, PA USA; 2 Department of Pediatrics, University of Pennsylvania Perelman School of Medicine, Philadelphia, PA USA; 3 Rowan-Virtua School of Osteopathic Medicine, Stratford, NJ USA

*“I will apply, for the benefit of the sick, all measures [that] are required, avoiding those twin traps of overtreatment and therapeutic nihilism.”* –Hippocratic Oath, Revised

Neonates have a higher incidence of sepsis than any other age group. The incidence of neonatal early-onset sepsis (EOS), a systemic bacterial or fungal infection that occurs within the first three days after birth, is approximately 1 in 1000 live births in the US.^1^ The clinical presentation of EOS is often non-specific and overlaps with delayed extrauterine transition and other neonatal disease entities, including the inherent physiologic instability related to prematurity.^2^ The consequences of EOS can be tragic; the fatality rate for term and late preterm neonates is approximately 1–3%, and survivors are at increased risk of several morbidities.^2^ Thus, not surprisingly, clinicians have a low threshold to evaluate neonates for EOS at birth and prescribe empiric antibiotics pending the results of blood and spinal fluid cultures. Microbial culture is the gold standard test for EOS and is highly sensitive when appropriately obtained.^2^ Expert guidance in the US encourages discontinuing empiric antibiotics if cultures are negative beyond a time threshold based on reported times to culture positivity, typically 24–48 h.^2^ Therefore, prescribing antibiotics beyond this threshold for ‘culture-negative sepsis’ should be a rarely used management strategy—an exception to the rule, reserved only for atypical cases. The reality, however, is that a substantial portion of neonatal antibiotic exposure results from prolonged treatment despite negative cultures.

In this issue of Pediatric Research, Dimopoulou et al. report the results of a secondary analysis of an international observational study of early antibiotic use among more than 750,000 term and late preterm neonates (≥34 weeks’ gestation) with suspected EOS across 13 centers in 11 high-income countries from 2014 to 2018.^3^ Of the 21,703 (2.9%) neonates who received intravenous antibiotics, more than one-third received prolonged treatment, defined as five or more days, in the setting of negative cultures (deemed culture-negative sepsis). Only 1.7% of neonates with suspected EOS treated with antibiotics had positive cultures. The incidence of culture-negative sepsis treated with prolonged antibiotics was more than 20 times that of culture-positive sepsis. Lastly, their findings highlight remarkable variability across institutions regarding the continuation of antibiotics when cultures were negative, with the incidence of culture-negative sepsis with prolonged treatment varying 11-fold.

Antibiotics are the most commonly prescribed discretionary medications in neonatal units and are most often prescribed empirically for suspected infection.^4^ Other drugs, too, are prescribed empirically to newborns while awaiting test results. For example, a prostaglandin infusion for a critically ill neonate who could have ductal-dependent congenital heart disease or phenobarbital for a neonate with suspected clinical seizures. It would be unusual, however, to continue these risky therapies when gold standard tests confirm the absence of the suspected disease. If the echocardiogram is normal, prostaglandin should be stopped. If the electroencephalogram confirms the absence of seizures, phenobarbital should be discontinued. Why, then, do neonatal clinicians in high-income countries with access to modern, advanced culture technology continue to prescribe prolonged courses of antibiotics when cultures are negative? Others have outlined the possible reasons for this persistent “culture” in neonatal medicine, including concerns about blood culture sensitivity with limited blood volume and after maternal/neonatal antibiotic exposures, and reliance on biomarkers of inflammation.^5^ These concerns have been countered or debunked, yet the current study highlights that this is a persistent international phenomenon. What can be done to move the needle forward to optimize antibiotic prescription, a mainstay of modern neonatal care, and more thoughtfully balance the risks and benefits?

First and foremost, we should all reflect on our approaches to cultural best practices, which are critical for accurate diagnosis and management of EOS (Table 1).^2,6–8^ Blood culture accuracy is volume-dependent; thus, obtaining an optimal volume of at least 1 mL per bottle *must be a priority*.^2^ Biomarkers such as complete blood cell counts and C-reactive protein are non-specific yet commonly used to diagnose culture-negative sepsis. Perhaps the blood volume used for these tests could be added to the blood culture bottle instead. Collecting more than one culture, carefully selecting culture sites, and adding anaerobic cultures can all increase the sensitivity and specificity of bacterial detection.^2,8^

**Table 1:** Best practices for blood cultures in neonates.

Practice	Recommendation
Timing of cultures	Blood cultures should be drawn prior to initiation of antibiotics
Site of blood draw	Peripheral venipuncture is the preferred method over catheter-based methods to prevent cross-contamination. Umbilical artery catheter is an acceptable alternative.
Volume of blood	A minimum of 1 mL per culture bottle is recommended to reduce false negatives
Number of cultures	Using two separate cultures can help distinguish between contamination versus true infection
Aerobic/anaerobic cultures	Using one aerobic and one anaerobic culture bottle can optimize the detection of facultative anaerobes, and also increases the sensitivity of detection of organisms
Culture technique	Aseptic techniques including hand hygiene, use of sterile gloves and drape, and proper disinfection techniques (e.g., waiting 30 s after applying disinfectant to draw blood) reduce the risk of contamination. Blood draw should be performed by more experienced personnel or phlebotomy team

Second, it is essential to understand the underlying causes of the clinical signs that lead clinicians to suspect EOS. *A wide range of conditions can mimic EOS*, including congenital defects, metabolic disorders, congenital viral infections, and other inflammatory conditions such as hypoxic ischemic encephalopathy and meconium aspiration syndrome.^7,9^ Early recognition of these conditions via clinical assessment, perinatal history, and other testing can help prevent the overuse of antibiotics. In the current study, no deaths among culture-negative cases with prolonged treatment were attributed to sepsis; more than half were attributed to malformations, and a third to perinatal asphyxia.

Third, we need more qualitative evidence about *why clinicians continue to prescribe prolonged courses of antibiotics to uninfected infants*. What are the thought processes that lead to these decisions? How can we overcome biases that influence our choices, such as commission bias and anchoring bias? Why are neonatal clinicians so untrusting of cultures, even when they are appropriately obtained? Are there liability concerns, and if so, are they well-founded? What role do non-physicians play in these decisions, including neonatal nurses, pharmacists, advanced practice providers, and parents/caretakers? Why do we discuss in-depth the risks and benefits of some treatments with families (i.e., medical therapy for patent ductus arteriosus, steroids for evolving chronic lung disease) but not for antibiotics? Lastly*, is there a role for continued antibiotics for critically ill infants with negative cultures*, and if so, how can we optimize such an approach or develop standards for future study? The benefits of continuing antibiotics for infected infants are clear, but the risks among the uninfected majority cannot be ignored and should be more transparent in daily practice.

Lastly, if there are indeed bacteria or other pathogens responsible for CN-EOS, *we must work tirelessly to identify them*. This is necessary to determine what we are treating, assess whether we are using the appropriate antibiotics, and evaluate effective treatment and resolution. How can we improve the detection of organ-specific infections? The diagnosis of neonatal pneumonia, for example, remains elusive. There is limited guidance on investigating other potential site-specific infections and also limited data on the adverse effects of antibiotic metabolism on injured organs (i.e., liver and kidneys in asphyxia) in the absence of infection. Non-culture-based technologies, including polymerase chain reaction-based methods, 16S rRNA sequencing, and microRNA analysis, are being studied.^10^ Each modality has pros and cons, but no currently licensed technologies in the US are more sensitive and specific than traditional cultures providing antibiotic susceptibilities. There is a pressing need to study these advanced technologies further to refine the diagnostic process of EOS and potentially identify novel prevention strategies.

Dimopoulou et al. highlight the persistent and widespread extent of prolonged antibiotic therapy for culture-negative neonatal EOS, including among centers with low rates of early antibiotic initiation. This suggests that even centers with optimized practices for antibiotic initiation struggle with deciding when to stop them. While intravenous antibiotics were unavailable in ancient Greece, the sentiment from Hippocrates remains relevant: overtreating neonates with antibiotics who are not infected will only cause harm. This international paradigm should continually be subject to challenge.
